# Clinical Presentation and Management Strategies in Intraorbital Foreign Bodies

**DOI:** 10.1155/2021/6645952

**Published:** 2021-03-30

**Authors:** Samreen Khanam, Ayushi Agarwal, Ruchi Goel, Neha Rathie, Akash Raut, Shweta Raghav, Sumit Kumar, Mohit Chhabra, Sonam Singh, Sushil Kumar

**Affiliations:** Guru Nanak Eye Centre, Maulana Azad Medical College, New Delhi, India

## Abstract

The authors present a retrospective, observational case study of seven patients, who presented with retained Intra-Orbital Foreign Bodies (IOrbFBs) following penetrating orbital injury at a tertiary eye hospital over a period of one year. Cases were reviewed for epidemiological features, mechanism of injury, nature of foreign body, clinical features, imaging modality, associated complications, management outcomes, and the final prognosis. The mean age of presentation was 27.43 years. Amongst the seven patients, two were children (aged <10 years). The male : female ratio was 4 : 3. Of the seven retained IOrbFBs, two were plastic, two wooden, and three metallic in nature (one gunshot injury, one ball projectile (commonly referred to as BB) injury, and one with knife). Two out of seven had no light perception at presentation. The periocular location of the foreign bodies was inferior in 4 cases and medial in 3 cases. Computed Tomography scan confirmed the diagnosis in five cases and Magnetic Resonance Imaging (MRI) was diagnostic in one. Surgical intervention was done in five cases, and two cases were managed conservatively. The authors conclude that favourable outcome can be achieved even without surgical removal in cases of inert metallic/inorganic IOrbFBs. The properties of plastic FBs can frequently render them invisible on imaging, or they may mimic chronic inflammatory conditions like tuberculosis. Long-standing wooden IOrbFBs evade identification radiologically due to prolonged hydration. The ultimate choice of intervention must be individualised, weighing the risks of retention against the risk of iatrogenic damage.

## 1. Introduction

Ocular trauma is a significant cause of blindness all over the world. Approximately 16% cases of orbital trauma are associated with a foreign body [1]. Intra-Orbital Foreign Bodies (IOrbFBs), though commonly encountered in routine ophthalmology practice, may have unusual presentations. They may occasionally accompany trivial trauma, such as in children while playing. The diagnosis and management of IOrbFBs are often a conundrum for most ophthalmologists. For a better understanding, the authors present their experience of seven cases of retained IOrbFBs and discuss their presentations as well as diagnostic and management dilemmas.

## 2. Methods

This is a retrospective analysis of seven patients, diagnosed with retained IOrbFBs following penetrating injury, who presented to a tertiary eye hospital in New Delhi, India, between December 2018-19. The epidemiological characteristics, clinical features, mechanism of injury, nature of foreign body, diagnostic imaging, associated complications, management outcome, and the long-term prognosis were evaluated.

## 3. Results

**Figure 1:**
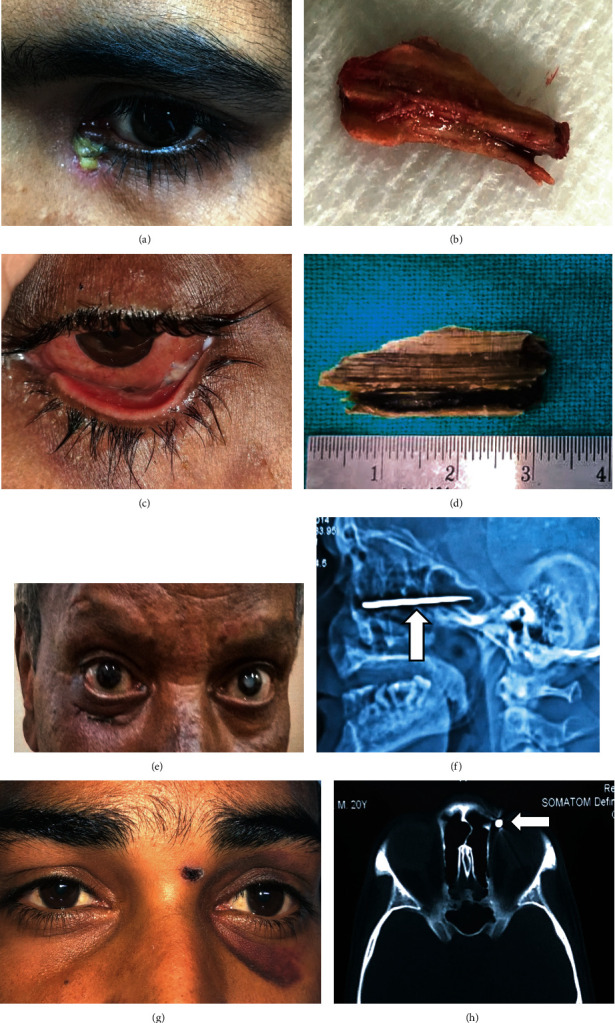
(a) Clinical photograph showing purulent discharge in the left medial canthal area and (b) retrieved wooden IOrbFB in case 6. (c) Clinical image showing orbital inflammation and (d) retrieved wooden IOrbFB in case 5. (e) Clinical picture depicting an entry wound below the right lower lid and (f) radiological image showing a linear metallic foreign body along the orbital floor in case 7. (g) An entry wound in the glabellar region and (h) noncontrast CT orbit (axial view) suggestive of a metallic foreign body post-BB injury in case 4.

Seven patients diagnosed with IOrbFB were studied (Table 1**)**. The mean age of presentation was 27.43 years. Amongst the seven patients, two were children (aged <10 years). The male : female ratio was 4 : 3. Of the seven retained IOrbFBs, two were plastic foreign bodies, two wooden, and three metallic in nature (one gunshot injury, one ball projectile (commonly referred to as BB) injury, and one injury with knife). The time of presentation varied from as early as three hours to a delayed presentation of three years. Only one of these patients was asymptomatic with a Best-Corrected Visual Acuity (BCVA) of 20/20 whereas one of them was diagnosed as Traumatic Orbital Apex Syndrome (TOAS) with a retained posterior foreign body (FB) and no light perception (NLP). Surgical intervention was recommended in six patients. The periocular location of the foreign body was inferior in 4 cases and medial in 3 cases. Three patients presented with features of inflammation including discharging sinuses. Traumatic optic neuropathy was seen in two patients (cases 1 and 2). Initial Computed Tomography (CT) scan could confirm the diagnosis in five cases whereas in one case, a repeat scan was helpful. MRI was diagnostic in one case (Figure 1).

### 3.1. Illustrative Case 1

**Figure 2:**
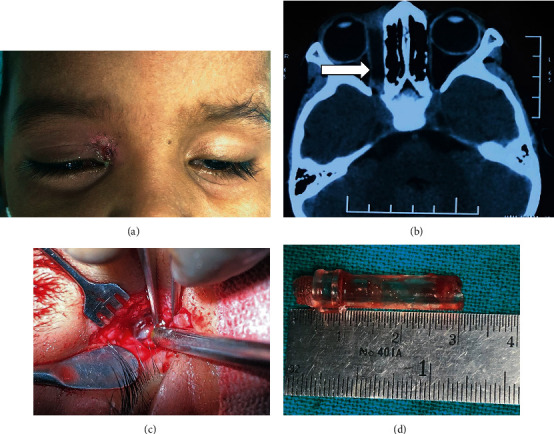
Illustrative case 1: (a) preoperative photograph revealing chronic discharging sinus in the right superomedial region; (b) NCCT orbit suggestive of a linear nonmetallic foreign body along the right medial wall of the orbit (arrow); (c) surgical exploration was performed; (d) retrieved plastic foreign body.

A 4-year-old male patient presented with features of orbital inflammation, impaired motility, and relative afferent pupillary defect (RAPD) in the right eye. Fundoscopy was suggestive of right optic atrophy. The left eye was unremarkable. A history of trauma to the right eye, while playing with a pen 5 months prior to presentation, was present. A provisional diagnosis of orbital cellulitis was made, and the patient was started on broad-spectrum antibiotics (linezolid 10 mg/kg IV q8 hr, metronidazole 10 mg/kg IV q8 hr and ceftriaxone 50 mg/kg IVq12 hr). Purulent discharge, sent for culture and sensitivity examination, from the superomedial aspect of the orbit was positive for *Staphylococcus aureus* and negative for AFB (Acid Fast Bacilli) staining. CT scan of the orbit was suggestive of soft tissue thickening with inflammatory changes more prominent in the extraconal, medial compartment of the right orbit. No abscess or foreign body could be identified. Empirical treatment resulted in clinical improvement. However, there was persistence of discharging sinus on the superomedial aspect of the right orbit. A review of the scan was requested, and a linear non-metallic FB running posteriorly along the medial orbital wall was identified. Surgical exploration was done, and a cylindrical plastic FB, measuring 30 mm × 3 mm, was successfully removed (Figure 2). The patient was continued on post-operative antibiotics. At 5-month follow-up, complete resolution of discharging sinuses and infection was achieved with no change in BCVA and fundoscopy.

### 3.2. Illustrative Case 2

**Figure 3:**
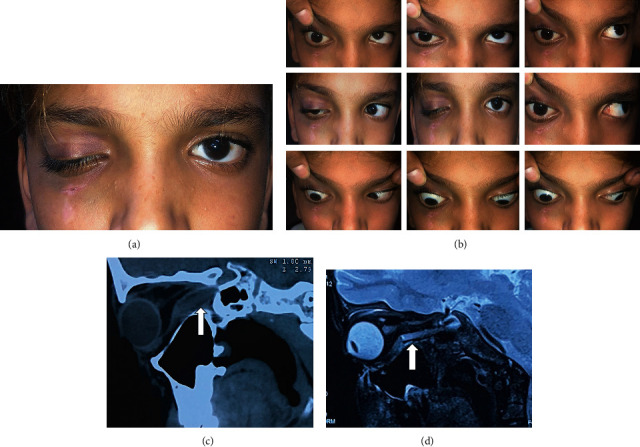
Illustrative case 2: (a, b) clinical examination showing ptosis, limitation of right extraocular movements, and an entry wound below the right lower lid; (c) NCCT orbit and (d) MRI orbit, sagittal view, reveal a nonmetallic object extending up to the apex (white arrow).

A 10-year-old male presented with sudden diminution of vision in the right eye for the past six days, following trauma from a ballpoint pen while playing at home. On examination, the right eye had NLP. An entry wound, 6 mm below the right lower lid, was noted. Right EOMs were limited in all gazes with ptosis, accompanied by hypoesthesia along the right forehead and upper lid region. A grade II RAPD with disc pallor was noted in the right eye. The rest of the examination was unremarkable. Initial Non-Contrast Computed Tomography (NCCT) scan was inconclusive. A repeat scan revealed a nonmetallic linear FB, 24 mm × 5 mm, lying in the posterior orbit. It was extraconal, posteriorly reaching up to the superior orbital fissure (SOF), inferiorly lifting the inferior rectus belly, and abutting the ipsilateral optic nerve. Fracture of the right orbital floor with fat herniation was also visualised. Magnetic Resonance Imaging (MRI) orbit further confirmed the CT findings. A Flash-Visual Evoked Potential (VEP) showed prolonged latency and low P-wave amplitude in the right eye. A confirmatory diagnosis of right TOAS was made. The patient was advised surgical removal of the IOrbFB due to its compressive effects and the FB being a potential source of infection. However, the patient's family refused surgery in view of increased perioperative morbidity and possibility of no significant gain in vision. Complete resolution of ptosis with full and free EOMs in the right eye was seen at a follow-up of 10 months with no change in BCVA (Figure 3).

### 3.3. Illustrative Case 3

**Figure 4:**
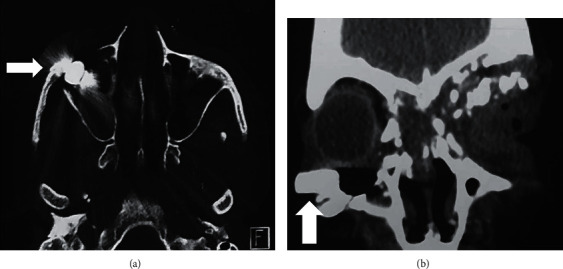
NCCT orbit: (a) axial view showing metallic density foreign body (white arrow); (b) coronal view showing metallic density foreign body in relation to the right orbital floor and in close proximity to the inferior rectus muscle (white arrow).

A 27-year-old female presented with painful swelling in both eyes. At presentation, she was disoriented to time, place, and person. Her husband gave a history of gunshot injury few hours back. On examination, circumferential limbal perforation with uveal tissue prolapse was noted in the left eye with no light perception (NLP). The right eye had lower lid swelling and a vision of 20/200 with vitreous hemorrhage and limited extraocular movements in upgaze. The patient was referred to the neurosurgery emergency department for adequate systemic management. NCCT scan of the head, orbit, and paranasal sinuses revealed a metallic density foreign body (suggestive of bullet) in relation to right orbital floor and in close proximity to the inferior rectus muscle with fat entrapment. It also showed fracture of the anterior and posterior wall of the frontal sinus, bilateral nasal bone and septum, and bilateral ethmoid along with fracture of the medial wall, lateral wall, and floor of the right orbit. Comminuted fracture of frontal bone on the left side involving the roof, medial wall, and floor of the left orbit was seen with no obvious entrapment of fat or muscle (Figure 4). The patient's left eye was eviscerated one day following presentation.

Her right eye was initailly planned for orbital floor reconstruction with foreign body removal.

A final visual acuity of 20/40 was, however, achieved with conservative management at one-year follow-up. Right extraocular movements were restored with no diplopia or significant enophthalmos.

### 3.4. Other Cases (Figure 1, Table 1)

#### 3.4.1. Case 4

A 30-year-old male with BB injuries at multiple sites and an entry wound at the glabella presented for ophthalmic evaluation within six hours of injury. An NCCT scan was suggestive of a metallic FB in the anterior orbit, adjacent to the left medial rectus. On surgical exploration, the FB could not be retrieved, and a coexisting lower canalicular injury was repaired with silicon tube intubation. The postoperative CT scan was suggestive of posterior migration of the foreign body. No further intervention was attempted. The BCVA was 20/20 OU, pre- as well as post-operatively. No extraocular motility limitations were seen.

#### 3.4.2. Case 5

A 35-year-old female presented with intermittent pain, discharge, and swelling in the left eye for three years with acute exacerbation of symptoms for 15 days. On examination, the left eye was hypotropic with associated chemosis, congestion, and limited extraocular movements in all gazes. The BCVA was 20/20 OD and 5/200 OS. The patient was started on empirical antibiotic therapy. Imaging was suggestive of a well-defined lesion in the posterior aspect of the left orbit between lateral and inferior recti with post-contrast enhancement. On surgical exploration, a flat wooden IOrbFB (3.8 cm × 0.5 cm) was obtained. On further questioning, the patient vaguely recalled an episode of trauma to the left eye while cutting firewood 3 years back. After completion of antibiotic therapy, there was complete resolution of inflammation and improvement in extraocular motility in the left eye along with restoration of BCVA to 20/20 OU.

#### 3.4.3. Case 6

A 36-year-old female presented with recurrent swelling in the left lower lid. There was an associated history of trauma to the left eye while cutting a wooden log 8 months back. The lacrimal apparatus was patent on syringing. Ultrasonography features were suggestive of left inferomedial orbital abscess. The abscess was drained surgically, and a wooden foreign body, measuring 2 cm × 1 cm × 0.3 cm, was retrieved.

#### 3.4.4. Case 7

A 50-year-old male presented with a history of physical assault with a knife to the right eye 1 day back. There was an entry wound 7 to 8 mm below the right lower lid, and the BCVA was 20/20 OD. No impairment of ocular motility was seen. NCCT orbit was suggestive of a linear metallic IOrbFB running along the right orbital floor which was successfully removed with surgical intervention.

## 4. Discussion

Cases of trauma involving the orbit and periorbital region are commonly encountered in clinical settings. Both trivial and severe cases may be accompanied by a FB retained in the orbit. However, the diagnosis and management of a retained IOrbFB are frequently a challenging exercise for the treating ophthalmologist. In patients who present early, clinical suspicion and appropriate imaging may be conclusive in most cases. However, a late presentation with a vague history of trivial trauma can prove to be particularly difficult. Studies have documented a time lapse of up to 50 years [2]. In our study, the time of presentation of one patient was as late as 3 years.

The presenting features of IOrbFBs include clinical signs of orbital cellulitis, orbital hematoma, proptosis, impaired motility, diplopia, visual field loss, orbital abscess, optic neuropathy/atrophy, SOF syndrome, or TOAS. It is imperative to enquire about history of trauma in such patients. The most common presenting features in our series were the signs of orbital inflammation (swelling, pain, abscess, sinuses), impaired motility, and disc pallor. IOrbFBs can settle in the orbit via transocular route and can have concurrent intraocular foreign body along with vitreous hemorrhage, retinal/choroidal detachment, uveitis, endophthalmitis, and panophthalmitis. The most interesting presentation in one of our cases was a plastic FB associated with chronic suppurative discharging sinus masquerading as a tuberculous osteomyelitis.

The nature of FB plays a decisive role in diagnosis and management. On the basis of composition, foreign bodies in the orbit can be metallic (iron, aluminium, lead), inorganic non-metallic (plastic, glass, rock, concrete), or organic ( wood, thorns, bones) [3]. A plain X-ray film is helpful in detecting a metallic FB, except aluminium due to its relative radiolucency [4]. Ultrasonography (USG), being noninvasive and inexpensive, is considered the preferred imaging modality for initial evaluation, especially for anterior IOrbFBs. A meticulously performed standardized USG (combined A-scan and B-scan) can ascertain the nature and location of the foreign body and the presence of associated ocular damage [5, 6]. However, it requires expertise and detection can be difficult due to masking by surrounding inflammatory and fibroadipose tissue [7]. In fact, USG has not been shown to yield significant results in most reports [2] and there is a high possibility of missing IOrbFB, as can be seen in case 6, where the FB was discovered only during the incision and drainage of the abscess.

CT scan, a more readily available imaging modality with a shorter acquisition time, is the investigation of choice [8]. Thin axial and coronal views allow accurate localization of high-density FB like metal or glass. Identification of low-density objects such as wood can be challenging. The minimum size of FB that can be detected by CT scan is 0.3 mm for metal and 0.5 mm for glass. Imaging of intraorbital wooden FB (IOrbWFB) is quite complex and depends upon several factors like size, type, and hydration. Dry wood appears hypodense and can frequently be confused with entrapped air as both have similar densities. On the other hand, fresh wood appears isodense with fat, posing a diagnostic dilemma. Prolonged retention of wood may render it iso- or even hyperdense as imbibition of fluid and granulation both increase over a period of time, resulting in higher attenuation [9–13]. In one of our patients, (case 5), the presentation was delayed for three years and difficulty was encountered in identifying the wood on the basis of MRI scan. This may be ascribed to hydration over a period of time. It has been observed that specific parameters like 4000 HU (Hounsfield unit) width/400 HU level are more effective for detection of IOrbWFB, and radiologists should be requested for these settings where retained wood is suspected [14]. If an object that is less dense than water is suspected, the parameters should be set to a level of -500 HU and a window width of 1500 HU to distinguish it from air [14].

MRI is considered the investigation of choice for organic FBs. In cases with a definitive history of injury with organic matter, it may be considered as the initial investigation of choice. For all other situations, initial CT to rule out metallic FB is advisable. An MRI scan allows for better assessment of soft tissue damage. Unlike CT, wood is usually hypointense relative to intraorbital fat on both T1- and T2-weighted sequences and does not show gadolinium enhancement. In T1-weighted images (T1-WI), a wooden FB gives a more uniform signal and is more discernible as compared to T2-weighted imaging (T2-WI). The possibility of getting motion artefacts is also higher in T2-WI [15, 16].

In our study, CT scan helped to clinch the diagnosis in 5 patients while MRI was the diagnostic requirement in only one case of wooden FB. Two of our patients had plastic IOrbFBs, in whom diagnostic difficulties were encountered during imaging. Initially missed on CT, the FB was identified on subsequent scans. This may be attributed to the highly variable radiolucency of plastic. In some cases, plastic has been seen to resemble bone, while in others, it has mimicked air [14, 17].

All patients with retained IOrbFBs should be given anti-tetanus prophylaxis and broad-spectrum antibiotics. For wooden IOrbFBs, initiation of empirical antifungal therapy has been advocated, owing to the frequent polymicrobial nature of the associated infection [18]. The decision to remove FB should be weighed against the risk of iatrogenic damage to the orbital structures. Retention of organic IOrbFB has a much higher rate of infection and inflammation than nonorganic IOrbFB and should be removed in all cases. Wooden IOrbFBs are notorious for fragmenting intraoperatively. One must be careful not to leave behind splinters/fragments in such cases. A postoperative MRI must be advised to look for residual fragments, thereby ensuring complete removal. Nonorganic IOrbFBs have a lower rate of infection, and the decision to remove must be based on their location and the associated complications. Anteriorly placed FBs are generally more easily removed. Metallic compounds containing copper can incite intense inflammatory reaction, and removal is, therefore, necessary. Iron-containing IOrbFBs may cause siderosis, and in cases where conservative management is chosen, the patient should be routinely assessed with electroretinography [19]. Removal of a metallic FB also eliminates the contraindication of MRI, if the latter is required in the future.

One patient in our study had injury with a BB pellet (case 4). It should be noted that orbital injury with a projectile metal such as a pellet or bullet can extend up to the cranial cavity. Shock waves from the projectile may cause chorioretinitis sclopetaria, even in the absence of direct trauma to the eyeball [20]. Air gun pellets are made of 0.5% antimony, while BB pellets are made of steel coated with zinc or copper. These are generally well-tolerated, as seen in our case. Bullets are heavier with faster velocities and are more likely to be associated with severe damage. Shots in shotguns are usually made of lead, and their retention in the orbit, theoretically, may cause lead poisoning, although any such case has not been reported [21].

Foreign objects at the orbital apex and those extending beyond the superior orbital fissure are frequently associated with optic neuropathy and permanent damage to the neurovascular structures. Owing to their proximity to the apex, they are likely to contribute to significant ocular morbidity. FBs at the apex may present as Superior Orbital Fissure syndrome (SOF), OAS, Cavernous Sinus Syndrome, or may be completely inert [22]. Surgical removal can be done via transcranial approach or a transnasal transethmoidal endoscopic approach that provides a safer and less invasive access to FBs lying in the extraconal medial compartment of the orbit [23]. There are no clear-cut guidelines for the management of posterior IOrbFBs. The authors believe that in cases where inorganic posterior IOrbFBs are inert, surgical retrieval can be deferred due to an increased risk of causing damage to the surrounding structures, including the vascular supply.

**Figure 5:**
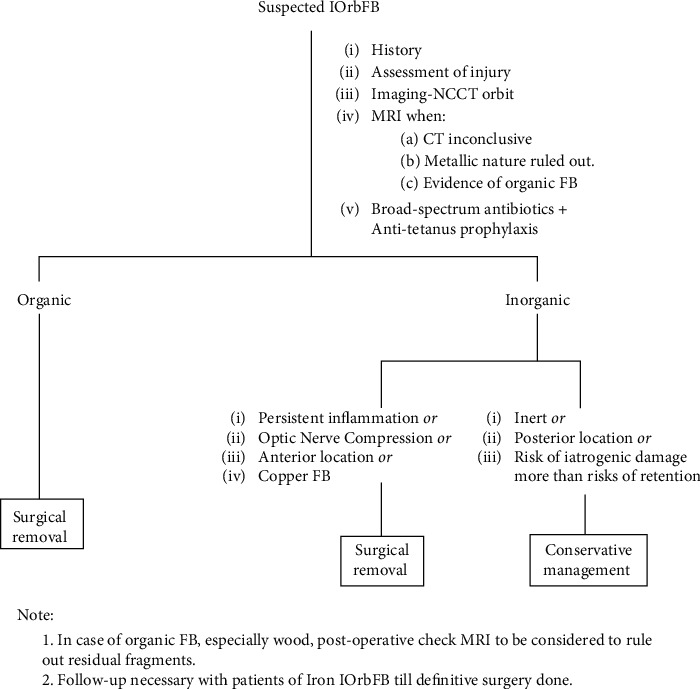
Approach to an intraorbital foreign body (IOrbFB).

Thus, in all cases of suspected IOrbFB, detailed examination of the eye and appropriate imaging should be performed to evaluate the site, size and nature of the FB, and the resultant damage incurred. Patients with IOrbFBs must be well-informed regarding the choices and the prognosis. Ultimately, inputs from both the ophthalmologist as well as the patient are important for the final decision. In the present study, both conservative and surgical management strategies have been adopted in different situations and based on these experiences, the authors conclude that every case behaves uniquely, and a tailored approach should be adopted by the surgeon (Figure 5).

## Figures and Tables

**Table 1:** Summary of clinical profile and management outcomes of cases with IOrbFBs.

No.	Age/sex	Nature of FB (location)	Mode of injury	Time between injury and presentation	Presenting features	Investigation	BCVA	Treatment/surgical findings	Status at last follow-up (duration)
1	4 y/M	Plastic FB (medial extending up to the apex)	Trauma while playing with pen	5 months	Right: Chronic discharging sinus at the medial canthal region with orbital cellulitis	CT: linear nonmetallic foreign body extending up to the posterior orbit. MRI: not done. Culture: *Staphylococcus aureus*	OD: not following light. Fundus suggestive of optic atrophy OS: following light. Refraction suggestive of hyperopia (+5D)	Intravenous antibiotics given Surgical removal of the 30 mm × 3 mm plastic FB done	OD: not following light EOM OU: full and free Discharging sinuses with orbital cellulitis resolved (5 months)
2	10 y/M	Plastic FB (inferior orbit, extending up to the apex)	Trauma while playing with pen	10 days	OD: traumatic superior orbital fissure syndrome with traumatic optic atrophy Entry wound seen 6 mm below the right lower lid margin	Initial CT inconclusive Repeat CT: nonmetallic linear IOrbFB (24 × 5 mm) between the floor and IR extending from the mid orbit to SOF, abutting the ipsilateral optic nerve MRI: FB extending up to the orbital apex abutting the optic nerve associated with right orbital floor fracture and fat herniation	OD: NLP OS: 20/20	Intravenous steroids given for TON Patient refused surgery in view of guarded visual prognosis	BCVA: OD NLP OS: 20/20 EOM OU: full and free Ptosis resolved Fundus OD: optic atrophy (10 months)
3	27 y/F	Metallic FB (inferior)	Gunshot	3 hours	OD: vitreous hemorrhage and limitation of movement in upgaze OS: circumferential limbal perforation with uveal tissue prolapse with NLP	CT: metallic FB in relation to the right orbital floor in close proximity to the IR along with a fracture of the right orbital floor, lateral wall, and medial wall. Comminuted fracture of frontal bone along with roof, medial wall, and floor of the left orbit. MRI: not done	OD: 20/200 OS-NLP	OD: surgery deferred Conservative management done OS: evisceration	BCVA: OD 20/40 OS: prosthetic eye EOM OD: full and free No significant enophthalmos noted (1 year)
4	30 y/M	Metallic FB (medial)	BB injury	6 hours	Referred for ophthalmic evaluation in view of BB injuries at multiple sites. Asymptomatic with entry wound at left glabellar area	CT: metallic FB in the anterior orbit adjacent to the medial rectus	OU: 20/20	Surgical exploration done. Intraoperative canalicular injury noted. Repair with silicon intubation done. FB could not be retrieved intraoperatively. Postoperative CT suggestive of posterior migration. No further surgical intervention done	Asymptomatic OU: 20/20 EOM OU: full and free Syringing: patent (6 months**)**
5	35 y/F	Wooden FB (anterior orbit extending up to the posterior orbit)	Undetermined	3 years	OS: diminution of vision associated with inferolateral swelling with limitation of extraocular movements in all gazes Left hypotropia with chemosis and congestion noted	CT: posterior orbital mass in the lateral half of posterior orbital fat. MRI: focal well-defined lesion in the posteroinferior aspect of the left orbit between LR and IR muscle with peripheral enhancement postcontrast suggestive of FB granuloma	OD: 20/20 OS: 5/200 PR accurate	Surgical exploration with histopathological examination A, 3.8 cm × 0.5 cm, wooden foreign body removed surgically	BCVA OU: 20/20 EOM OD: full and free OS: improvement in extraocular movements. Left hypotropia present. No diplopia seen Complete resolution of inflammatory signs (2 months)
6	36 y/F	Wooden FB (medial orbit)	Trauma while cutting wooden log	8 months	Left lower lid recurrent abscess Syringing patent	USG: inferior orbital abscess. CT/MRI: not done Culture: sterile	OU: 20/20	Surgical exploration: abscess drainage done. FB found and removed. Postoperative MRI showed no evidence of IOrbFB	BCVA OU: 20/20 EOM OU: full and free Complete resolution of signs and symptoms (1 year)
7	50 y/M	Metallic FB (inferior orbit)	Physical assault with a knife	1 day	OD: pain with entry wound 7-8 mm below the right lower lid margin OS: mature cataract	X-ray and NCCT orbit: suggestive of a linear metallic FB running along the right orbital floor	OD: 20/20 OS: PL positive PR accurate	Successful surgical removal of the linear foreign body (knife) OS: cataract surgery done a few days later	BCVA OD: 20/20 OS: 20/20 (8 months)

FB: foreign body; BCVA: best-corrected visual acuity; EOM: extraocular muscles; NLP: no light perception; CT: computed tomography; MRI: magnetic resonance imaging; PR: projection of rays; IR: inferior rectus; LR: lateral rectus.

## Data Availability

The data used to support the findings of this study are included in the article.
